# 小细胞肺癌与TNM分期

**DOI:** 10.3779/j.issn.1009-3419.2016.06.22

**Published:** 2016-06-20

**Authors:** 克能 陈

**Affiliations:** 100142 北京，北京大学肿瘤医院 Department of Thoracic Surgery, Beijing Cancer Hospital, Beijing 100142, China

**Keywords:** 小细胞肺癌, 外科治疗, TNM分期, Small cell lung cancer, Surgery, TNM staging

## Abstract

小细胞肺癌的临床侵袭性强，易于转移，因而长期以来认为手术疗效较差，治疗方法主要是放化疗，因此分期手段也以美国退伍军人肺癌协会的局限期和广泛期为主。随着肺癌分期手段的进步，分期的准确性进一步提高。大量回顾性的资料表明，早期小细胞肺癌的手术疗效不亚于非小细胞肺癌。而对拟手术的小细胞肺癌的分期也应采用现代更精确的TNM分期。

## Abstract (en)

Since the characeristics of high invasiveness and metastatic potential of small cell lung cancer, it has long been considered to be associated with poor clinical outcome, especially after surgery. The chemo-radiation, therefore, is the mainstream modality, and its staging relied on American Vterans Aministration system, a simple system mainly from radiation oncologists' idea. With the advances of staging methods in lung cancer, the accuracy of TNM staging has been increasing. More and more retrospective study results demonstrated that the efficacy of surgical intervention for early stage small cell lung cancer was not inferior to that for non-small cell lung cancer. Moreover, modern and precise TNM staging system should be adopted towards surgical candidates of small cell lung cancer.

肺癌的成功治疗源于1933年的左全肺切除术^[1]^，而人们认识到肺癌在组织学上可分为非小细胞肺癌（non-small cell lung cancer, NSCLC）与小细胞肺癌（small cell lung cancer, SCLC）已是至少30年之后的事情^[2]^。因此，可以说外科治疗SCLC经历了因认识不足而造成结果不理想的混杂阶段、适应证不清晰的失败阶段与现代认识下的成功阶段。在这一“混乱→失败→成功”的过程中，严格的TNM分期诊断的重要性进一步凸显。然而遗憾的是，SCLC的分期系统迄今仍并行着不同的方法。这就一定程度上给肺癌工作者，尤其是外科医生造成了不少的混乱。本文就此做一综述，供大家参考。

## 1 SCLC概念的由来及其演变

自1933年肺切除术成功运用于肺癌治疗以来^[1]^，手术曾一度成为SCLC的首选甚至是唯一治疗，人们也并未意识到有何不妥，但术后患者疗效差异巨大的现象，使得大家开始思考是否另有其他的影响因素。直到1962年将肺癌从病理学上分为SCLC与NSCLC才解决了这一问题^[2]^。SCLC来源于神经内分泌细胞，是具有生长速度快、恶性程度高且易发生纵隔淋巴结及远处转移、手术治疗疗效较差等特点的一组特殊类型的肺癌，可能不适于手术治疗。如上事实呼唤了两项临床随机研究，其一，1973年英国医学研究协会（Medical Research Council, MRC）对SCLC手术和放疗进行了随机研究，手术组71例，放疗组74例，中位生存期分别为199天和300天（*P*=0.04）；2年生存率分别为4%（3例），10%（7例）；5年时手术组生存仅1例，放疗组生存3例，随机分配至手术组的1例患者终因肺功能差而采取了姑息放疗；10年时手术组无生存，放疗组仍有3例生存。因此，研究者认为，与放疗相比，手术并不适用于SCLC的治疗^[3]^。其二，1994年肺癌研究组（Lung Cancer Study Group）对以环磷酰胺+多柔比星+长春新碱方案化疗序贯脑、胸部放疗后，加与不加手术做了研究，手术组70例，非手术组76例，中位生存期分别为15.4个月和18.6个月，2年生存率均为20%^[4]^。认为肺切除在SCLC的多学科综合治疗中并无益处。以上研究确立了不宜外科治疗SCLC的观念，自此，主流外科淡出了SCLC的治疗领域。

## 2 肺癌TNM分期的由来

TNM分期是当今实体恶性肿瘤诊治的重要内容，在学术交流、诊断、治疗、预后等方面不可或缺，已广泛应用于绝大多数实体恶性肿瘤的临床实践^[5]^。在此，我们简要回顾一下TNM分期的由来及其在肺癌临床中的实践。这里不得不提及的是法国肿瘤外科学家Pierre Denoix（1912-1990），其毕生最辉煌的成就就是发明了肿瘤的TNM分期系统（1943-1952），并由此于1973年-1978年间出任国际抗癌联盟（Union for International Cancer Control, UICC）主席^[6]^。UICC始于1934年的法国，是目前被肿瘤学界广泛认可的学术组织，负责恶性肿瘤TNM分期系统的定期更新。TNM分期概念于1954年第一次被接受，纳入了包括1966年提出的肺癌分期在内的共计23种肿瘤，并于1960年-1968年首次以出版物发行。与此同时，美国于1959年成立了相似功能的机构，并于1980年正式更名为美国癌症联合会（American Joint Committee on Cancer, AJCC），至此AJCC与UICC形成了一并共存的格局，均致力于实体肿瘤的分期，但当时两者均无数据支持，仅以专家共识推出，两者的分期内容虽有争议，但极为相似^[7]^。好在1969年在多伦多，两个来自不同地域的机构就恶性肿瘤的分期达成了共识，即现今被大家熟知并延用的UICC/AJCC分期系统。TNM分期至2009年已出版第7版，2017年将陆续出版第8版分期。肺癌TNM分期在第5、6版才出现小样本数据支撑，及至2009年第7版时，肺癌的分期改良已建立在多达十万以上的数据分析之上^[8]^。

## 3 SCLC的VALG分期系统由来

由于不利于手术的临床研究结果的公布^[3, 4]^，大家普遍认为SCLC在诊断时就已是全身性疾病，而治疗须以全身化疗为主，辅以局部放疗，因此由放疗科医师主导的独立于TNM分期之外的美国退伍军人肺癌协会（Veterans Administration Lung Study Group, VALG）分期系统经历从1977年开始的酝酿阶段，至1984年达成专家共识，进入成熟阶段并引入临床应用，该分期将SCLC简略地分为局限期（limited disease, LD）与广泛期（extensive disease, ED），前者定义为病变局限于一侧胸腔包括伴有对侧纵隔及双侧锁骨上淋巴结有转移者，以及同侧有胸水者，暗含一个可承受的完整放射野，反之，病变超出上述范围者均定义为广泛期^[9]^。

VALG分期系统的问世有如下背景：①SCLC生物学行为高度恶性，侵袭性极强，极易出现播散。因此，多数SCLC诊断时即为全身不可治愈性的疾病。②TNM分期需分别对局部肿瘤、区域淋巴结及远处转移脏器分别评估，分期方法相对繁琐，不适合放疗科医生的应用。③SCLC只要出现单个脏器转移，其治疗以全身化疗为主。更多的、更详细的分期检查手段对制定治疗方案并无帮助，反而增加额外的医疗费用。

## 4 外科治疗SCLC地位的再肯定

虽然临床试验表明手术不适用于SCLC的治疗，但下列原因造就了SCLC外科治疗的持续存在，并在上世纪90年代达到高峰，约47%的SCLC被手术切除，直至2005年手术病例才降至全部SCLC的16%，其原因如下：①机会性切除，即术前无病理而术后病理诊断为SCLC。②部分外科医生不遵循肿瘤学“原则”，而坚持手术切除SCLC。③部分SCLC系混合肿瘤，即使术前获得病理诊断，也难以在活检小标本上区别纯SCLC和混合性SCLC，因而得到手术治疗。鉴于如上原因，若从肺癌手术成功算起，临床上已积累了相当多SCLC的外科治疗结果。美国SEER数据库为再评估SCLC手术疗效提供了客观上的便利。

进入2010年后有多篇来自SEER数据库的大宗回顾性研究涉及这一课题，首先，是美国纽约州立大学南部医学中心的放疗科医生Schreiber等^[10]^于2010年发表于*Cancer*上的大样本回顾研究，该研究分析了1988年-2002年SEER数据库中T1-2NX-0以及T3-4NX-0的LD-SCLC患者的资料，共计14, 179例，其中863例患者接受了手术治疗，以T1、T2期患者为主，其中T1-2NX-0患者中位生存期由非手术治疗的15个月提升至42个月（*P* < 0.001），T3-4NX-0患者中位生存期由非手术治疗的12个月提高至22个月（*P* < 0.001）；肺叶切除术的预后最佳（*P* < 0.001），T1-2NX-0患者中位生存期达65个月、5年生存率52.6%，T3-4NX-0SCLC肺叶切除者中位生存期25个月，5年生存率31.8%。进一步分层发现，2, 251例N0患者中，435例手术治疗者的中位生存期40个月，优于非手术者的15个月（*P* < 0.001）；802例N0患者中164例手术治疗者中位生存期29个月，优于非手术者的14个月（*P* < 0.001）；7, 974例N2患者中187例手术治疗者中位生存期19个月，同样优于非手术者的12个月（*P* < 0.001）。因此，得出手术治疗LD-SCLC优于放化疗的结论。

几乎是同一时期，由不同的外科医师发表了2篇来自SEER数据库的分析，分别是：①Varlotto等^[11]^回顾1988年至2005年SEER数据库共计2, 214例LD-SCLC的治疗情况，发现单纯肺叶或扩大切除术患者的中位生存期50个月，优于接受单纯亚肺叶切除术患者的30个月（*P*=0.006），更优于接受单纯放疗患者的20个月（*P* < 0.000, 1）；单纯亚肺叶切除术者的中位生存期也优于单纯放疗（*P*=0.002）。是否加术后放疗的患者中位生存期差异无统计学意义（30个月对28个月，*P*=0.6）。该研究同样得出手术治疗LD-SCLC优于非手术治疗的结论。②2012年，Benny等^[12]^回顾性分析1988年至2007年间美国SEER数据库中诊断为Ⅰ期-Ⅱ期的3, 566例SCLC患者，其中行手术者为895例（25.1%）。手术方式包括楔形切除251例（28%），肺叶切除或全肺切除为637例（71.2%），术式不明确者7例（0.78%）。生存分析显示中位生存期手术组为34个月，非手术组为16个月（*P* < 0.001）。肺叶切除或全肺切除组的中位生存期为39个月，明显优于楔形切除组的28个月（*P*=0.001）。尽管如此，楔形切除组的生存仍优于非手术组（*P* < 0.001）。因此，研究者认为Ⅰ期及Ⅱ期SCLC手术治疗可明显获益。

## 5 现代外科治疗SCLC需更严格的TNM分期

分析否定外科不宜治疗SCLC的早期临床研究存在以下问题：①为中心型肺癌而行全肺切除术；②仅有48%的患者为“完全切除”，34%为探查或姑息手术，有18%的患者因各种原因最终放弃手术治疗；③分期诊断手段受限，无CT、MRI，更无PET等检查进行分期，很难排除远处转移；④无纵隔淋巴结分期手段，如纵隔镜、超声内镜引导下的透壁穿刺活检（endobronchial ultrasound-guided transbronchial needle aspiration, EBUS-TBNA）或TBNA等^[3]^；⑤1994年的随机对照临床试验，无淋巴结转移者仅占49%（29/70），且并未对pN0患者预后进行分层研究，化疗方案选用的也并非SCLC最佳的EP方案^[4]^。

1973年至2007年SCLC的分期主要采用VALG局限期和广泛期的两分法。该方法极其不适用于考虑手术治疗的SCLC患者，对于考虑手术治疗的SCLC患者应遵循NSCLC分期的TNM系统，并应更为严格。Shepherd等^[13]^对12, 620例SCLC患者中能按TNM分期者仅有8, 088例，按7版TNM分期的研究, 结果显示349例（2.8%）接受了手术治疗的患者中pⅠa期、pⅠb期、pⅡa期、pⅡb期、pⅢa期和pⅢb期的5年生存率分别为56%、57%、38%、40%、12%和0。TNM分期用于可切除的SCLC，按TNM分期外科治疗的结果其5年生存率并不差于NSCLC。该研究认为当评估可手术治疗的SCLC时，TNM分期比LD/ED两分法分期更有用，pⅠa期与pⅠb期以及pⅡa期与pⅡb期手术切除的预后相似，说明将来应寻找更敏感的方法加以区别。Nicholson等^[14]^对5, 002例SCLC患者按照第8版分期的研究，结果显示其中4, 848例患者具有临床TNM分期，582例患者具有病理TNM分期。无论按照第7版或第8版的分期标准，SCLC患者生存率均随T分期和N分期的升高而逐级下降，且差异具有显著性意义（*P* < 0.001）。该研究还发现，单发脑转移的SCLC患者预后好于其他部位单发转移或多发转移者。研究者认为SCLC同样适用于TNM分期并能判断预后。

综上所述，SCLC治疗前评估，尤其是患者淋巴结转移情况的术前评估，是决定LD-SCLC患者能否从手术治疗中获益的重要因素。虽然从2010年以后，SEER数据中对SCLC治疗有利的正向数据问世，但SCLC高度恶性易转移、全身性疾病的实质和对化疗敏感等特点并未被否认。同时，SEER数据仍为回顾性研究，目前并无大宗现代术前分期背景下的前瞻性随机研究。所以在呼吁开展SCLC相关外科治疗与非外科治疗前瞻性随机研究的同时，应审慎评估SCLC的手术适应证，至少应强化术前检查，以尽量避免术前可能已有的全身或纵隔转移，这就对LD-SCLC的术前分期与检查提出了更高的要求。
